# Ceramide-1-phosphate transfer protein promotes sphingolipid reorientation needed for binding during membrane interaction

**DOI:** 10.1016/j.jlr.2021.100151

**Published:** 2021-11-20

**Authors:** Yong-Guang Gao, Jeffrey McDonald, Lucy Malinina, Dinshaw J. Patel, Rhoderick E. Brown

**Affiliations:** 1 Hormel Institute, University of Minnesota, Austin, MN, USA; 2 Structural Biology Program, Memorial Sloan Kettering Cancer Center, New York, NY, USA

**Keywords:** lipid-transfer proteins, membranes/physical chemistry, membranes/model, protein structure, human glycolipid transfer protein (GLTP) superfamily, Arabidopsis accelerated cell death (ACD11) protein, peripheral membrane penetration depth, ceramide-1-phosphate binding, GLTP-fold alpha-helix induced lipid reorientation, ACD11, Arabidopsis accelerated cell death- CPTP ortholog, C1P, ceramide-1-phosphate, CHARMM, Chemistry at HARvard Macromolecular Mechanics, CPTP, ceramide-1-phosphate transfer protein, FAPP2, phosphatidylinositol-4-phosphate adapter protein-2, GLTP, glycolipid transfer protein, LTP, lipid transfer protein, OPM, Orientation of Proteins in Membranes, PH, pleckstrin homology, PPM, Positions of Proteins in Membranes, SL, sphingolipid.

## Abstract

Lipid transfer proteins acquire and release their lipid cargoes by interacting transiently with source and destination biomembranes. In the GlycoLipid Transfer Protein (GLTP) superfamily, the two-layer all-α-helical GLTP-fold defines proteins that specifically target sphingolipids (SLs) containing either sugar or phosphate headgroups via their conserved but evolutionarily-modified SL recognitions centers. Despite comprehensive structural insights provided by X-ray crystallography, the conformational dynamics associated with membrane interaction and SL uptake/release by GLTP superfamily members have remained unknown. Herein, we report insights gained from molecular dynamics (MD) simulations into the conformational dynamics that enable ceramide-1-phosphate transfer proteins (CPTPs) to acquire and deliver ceramide-1-phosphate (C1P) during interaction with 1-palmitoyl-2-oleoyl phosphatidylcholine bilayers. The focus on CPTP reflects this protein’s involvement in regulating pro-inflammatory eicosanoid production and autophagy-dependent inflammasome assembly that drives interleukin (IL-1β and IL-18) production and release by surveillance cells. We found that membrane penetration by CPTP involved α-6 helix and the α-2 helix N-terminal region, was confined to one bilayer leaflet, and was relatively shallow. Large-scale dynamic conformational changes were minimal for CPTP during membrane interaction or C1P uptake except for the α-3/α-4 helices connecting loop, which is located near the membrane interface and interacts with certain phosphoinositide headgroups. Apart from functioning as a shallow membrane-docking element, α-6 helix was found to adeptly reorient membrane lipids to help guide C1P hydrocarbon chain insertion into the interior hydrophobic pocket of the SL binding site.These findings support a proposed ‘hydrocarbon chain-first’ mechanism for C1P uptake, in contrast to the ‘lipid polar headgroup-first’ uptake used by most lipid-transfer proteins.

Nonvesicular sphingolipid (SL) trafficking in cells is mediated by lipid-transfer proteins (LTPs) that acquire and release their specific SL cargoes via transient interaction with source and destination biomembranes. In the GlycoLipid Transfer Protein (GLTP) superfamily, two families of cytosolic proteins exist that target ceramide-based SLs containing either phosphate or sugar headgroups. Phosphate headgroup SL-specific family members include human ceramide-1-phosphate (C1P) transfer protein (CPTP; 214 a.a.) and the plant CPTP ortholog, ACD11 (206 a.a.); whereas glyco-headgroup SL-specific members include human GLTP (209 a.a.), fungal HET-C2 (208 a.a.), and human phosphatidylinositol-4-phosphate adapter protein-2 (FAPP2; 519 a.a.). Divergent evolutionary modifications to the two-layer, all-α-helical GLTP-fold dictate SL polar headgroup selectivity via differing sequence motifs (DxxxK…RxxxRxxR…H in CPTPs; DxxxNxxK…WxxR…H in GLTPs) but conserved 3D topology to form surface-localized SL headgroup recognition centers, which connect to an internal hydrophobic pocket that accommodates the SL hydrocarbon chains. The targeting of the ceramide moiety originates from the highly conserved spatial locations of Asp and His in the SL recognition center that bind in pincher-like fashion with the ceramide amide group (1, 2, 3, 4, 5, 6, 7, 8, 9, 10, 11, 12).

Mechanistically, small single-domain GLTP superfamily members, such as CPTP and GLTP, function as ‘soluble shuttles’ that transit between membranes to acquire and deliver their lipid cargo (4, 5, 13, 14). The only known exception is for the much larger multi-domain FAPP2, which uses a N-terminal pleckstrin homology (PH) domain to target and dock with membranes containing phosphatidylinositol 4-phosphate whereas its C-terminal glycolipid-binding GLTP domain transits back and forth to transfer glycosphingolipids to nearby destination membranes (9, 12, 15, 16, 17). As such, FAPP2 can be classified as an LTP anchored at membrane contact sites. In this regard, FAPP2 is similar to the non-GLTP superfamily member, ceramide transfer protein (624 a.a.). Like FAPP2, ceramide transfer protein relies an N-terminal PH domain to target phosphatidylinositol 4-phosphate and help guide interaction with specific membranes but uses a C-terminal StAR-related lipid transfer domain to bind ceramide during the back and forth shuttling needed to redistribute ceramides between closely apposed membranes (18, 19). So, although the issue of what guides FAPP2 to specific membranes is reasonably well established, only recently have insights begun to emerge for small, single-domain GLTP superfamily members. CPTP has been shown to harbor-specific sequence motifs within its membrane interaction region that interact with select cytoplasm-facing anionic phosphoglycerides (e.g., phosphatidylserine and specific phosphoinositides) to enhance operating efficiency by optimizing protein orientation during membrane docking (20, 21).

Insights into the in vivo functional and pathophysiological roles of GLTP superfamily members also have recently begun to emerge (10, 11, 14, 22, 23). The advances implicate human GLTP superfamily member expression in the regulation of the following: *i*) cell-cycle arrest and necroptosis induction in certain colon cancer cell lines (23); *ii*) neuronal myelination and Niemann-Pick type-C disease (24); *iii*) hepatitis C viral infectivity (25); *iv*) cytoplasmic phospholipase A2α activation associated with pro-inflammatory eicosanoid production (10); *v*) autophagy induction and inflammasome assembly that drive surveillance cell release of interleukin-1β and interleukin-18 inflammatory cytokines (22). Owing to the emerging roles of C1P-transferring GLTP superfamily members in pathophysiological situations involving inflammation and autophagy, a need exists to better understand the functional dynamics that enable membrane interaction, SL uptake/release, and the atypical-bound lipid orientation associated with the GLTP protein superfamily. Herein, we report insights gained into the associated dynamic conformational features of human CPTP using molecular dynamics (MD) simulations.

## Materials and methods

### Preparation of membrane-embedded CPTP

CPTP orientation and position in the membrane was initially estimated using the Orientation of Proteins in Membranes (OPM) program (26, 27, 28). The protein was then embedded into a lipid bilayer using the Chemistry at HARvard Macromolecular Mechanics (CHARMM)-GUI membrane builder (29, 30, 31, 32). Both the outer and inner membrane leaflets were composed predominantly of POPC but sometimes contained C1P (2.5 mol%) and/or phosphatidylinositol 4,5 bisphosphate (PI-(4,5)P_2_) (10 mol%). The membrane/protein system was fully solvated with TIP3P water (33) and buffered in 150 mM NaCl to keep the system neutral. The resulting system (∼39,500 atoms) that included 93 POPC molecules was contained in a 60 × 60 × 120 Å^3^ simulation box. CPTP embedding in one side of the bilayer resulted in the opposite bilayer side without protein having 53 POPC molecules with average surface areas of ∼68.6 Å^2^/molecule. This value mimics the lipid packing in fluid-phase phosphatidylcholine (PC) bilayer vesicles determined by X-ray and neutron diffraction studies (34, 35).

### Molecular dynamics simulations

MD simulations were carried out with NAMD2.9 (36) using CHARMM36m (37) parameters set to model the system. NAMD is a parallel, object-oriented molecular dynamics code designed for high-performance simulation of large biomolecular systems and is CHARMM file-compatible. CHARMM3m provides improved accuracy in generating force fields for proteins polypeptide backbone conformational ensembles (37) and is compatible with CHARMM-GUI 6 force fields that enable interactive building of all-atom protein/membrane or membrane-only systems for MD simulation through an automated optimized process. The fast Fourier transform grid information of the Particle-mesh Ewald method is generated automatically. Particle-mesh Ewald method has become a standard method for treating long-range electrostatics in molecular simulations and is widely used because of its high efficiency, which stems from the use of fast Fourier transforms. Simulation systems were subjected to Langevin dynamics and the Nosé-Hoover Langevin piston method (38, 39) to maintain constant pressure (1.01325 Pa = 1 atm) and temperature (T = 303.15 K) (NPT ensemble). The simulations were first optimized by the standard CHARMM-GUI 6-step equilibration and minimization, as illustrated in supplemental Fig. S1, before initiating 300-ns unrestrained production simulations. Visual molecular dynamics software was used to display, analyze, and animate trajectories (40).

### Clustering analysis

For the 300 ns trajectory of the protein/membrane system, the clustering was carried out using TTClust (41). Ten classes were obtained, and a representative conformation from each class was generated automatically for subsequent analysis.

### Analysis of protein ligand interaction

The protein-ligand interaction profiler Server (42) was used to analyze the interaction between protein and its ligands. The Visual molecular dynamics tool ContactFreq.tcl was used to calculate the contact between ligand and protein residues from the MD simulations with contact being defined as <4 Å distance.

## Results

Current insights into the topology of CPTP membrane interaction are based almost entirely on comparisons with GLTP because GLTP has been more comprehensively characterized, and both proteins share the all α-helical GLTP-fold as their fundamental structural platform (1, 2, 3, 4, 5, 6, 7, 8, 9, 10). In the case of GLTP, functional mutation analyses and topology modeling using the OPM and Positions of Proteins in Membranes (PPM) programs have yielded insights into the protein’s spatial orientation and positioning during interaction with the lipid bilayer (4, 6, 10, 43, 44, 45, 46, 47, 48, 49, 50). In CPTP, the same general surface region has been implicated in membrane interaction as that of GLTP, but other details remain unclear (5, 10).

### CPTP membrane interaction during MD simulation

OPM/PPM modeling relies on an implicit solvation model for the lipid bilayer to approximate protein positioning and calculate membrane penetration (26, 27, 28) but does not provide insights into the dynamic changes experienced by CPTP during membrane-lipid interaction or lipid-cargo binding. Thus, we applied MD simulations to CPTP and analyzed four states associated with the protein’s intermembrane transfer function of C1P. The four CPTP states involve shuttling of either the apo- or holo-forms through the aqueous phase between membrane-lipid bilayers and interaction of either the apo- or holo-forms with membrane-lipid bilayers to achieve C1P uptake or release (supplemental Fig. S2). These states reflect mechanistic stages obtained for GLTP by kinetic, thermodynamic, and equilibrium binding analyses (43, 50) as well as X-ray crystallography (1, 2, 3, 4, 5, 6, 7, 8).

**Fig. 1:**
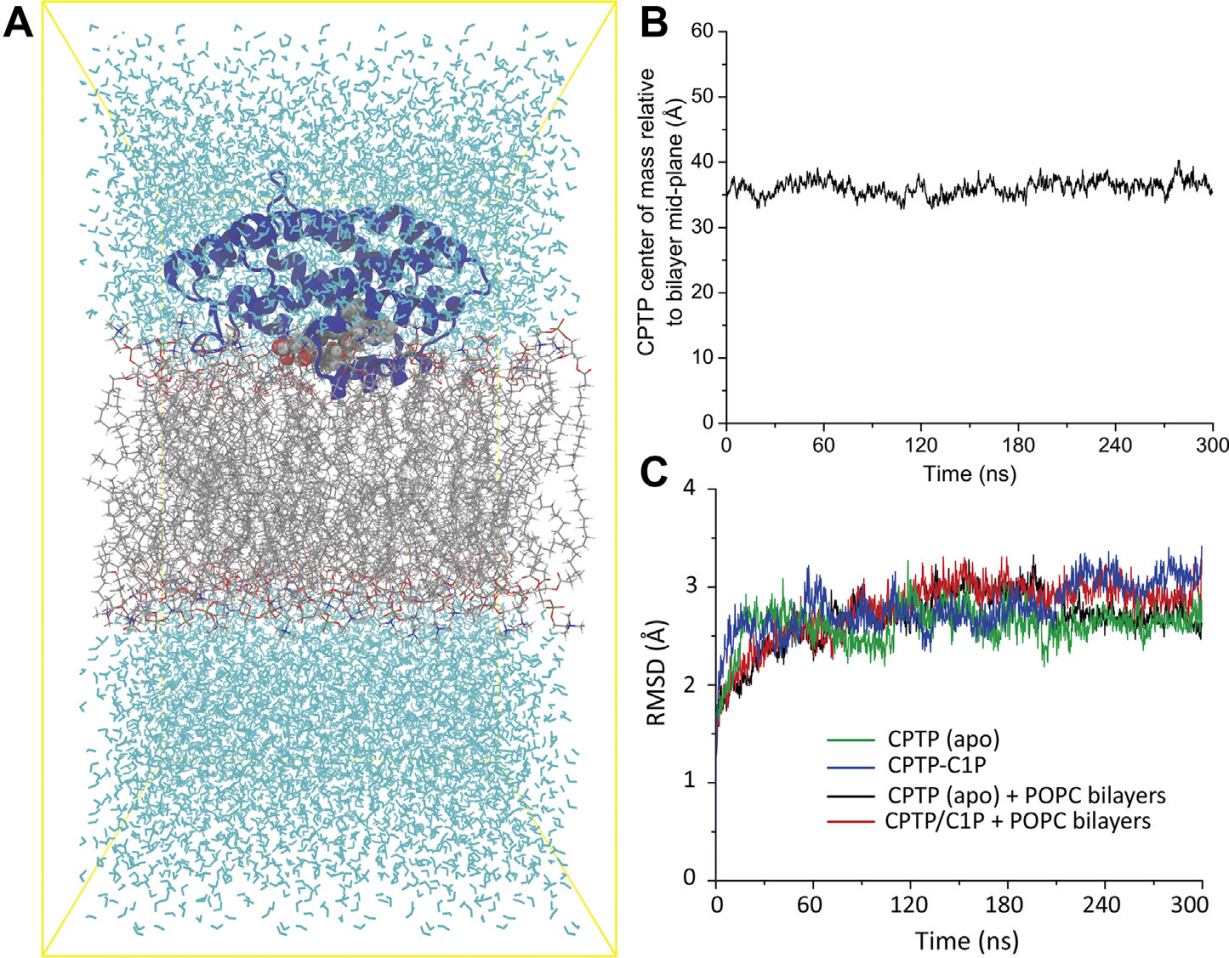
MD simulation of CPTP. A: The initial configuration for the simulation of the system. The protein is represented in its secondary structure (dark blue). C1P (space-filling) and POPC (stick) hydrocarbon chains are gray with oxygens colored red; nitrogen, blue; and phosphate, orange. The water molecules are cyan. The whole system is boxed with yellow lines. B: CPTP center of mass location relative to POPC bilayer mid-plane versus simulation time. *Y*-axis value of 0 Å = bilayer mid-plane and value of ∼20.5 Å = phosphate in POPC headgroup. C: Root-mean-square deviations (RMSD) of CPTP backbone versus simulation time for each CPTP state shown in supplemental Fig. S2.

Before initiation of the 300 ns MD simulations, the starting conditions for protein orientation and penetration depth into the POPC bilayer were set up using OPM/PPM modeling and then were optimized by the standard CHARMM-GUI 6-step equilibration and minimization. Figure 1A provides a resulting snapshot of CPTP with bound C1P interacting with the POPC bilayer. Tracking of the position of CPTP’s center of mass with respect to the POPC bilayer mid-plane indicated that the protein’s shallow penetration depth into the POPC bilayer remains nearly unchanged throughout the 300 ns MD simulation (Fig. 1B). Monitoring of the root-mean-square deviation(RMSD) values of the protein backbone atoms revealed somewhat similar profiles (Fig. 1C) for each of the four CPTP states associated with membrane interaction and C1P uptake/release (supplemental Fig. S2). Root mean square deviation values increased slightly (1.0–1.5 Å) over the initial 60 ns but then exhibited mostly small fluctuations (≤0.5 Å) for the remaining 240 ns, thus validating the stability of each protein state. Neither C1P binding to CPTP nor CPTP interaction with the POPC bilayer in the apo- or holo-forms triggered major changes in the overall dynamics and stability of CPTP.

**Fig. 2:**
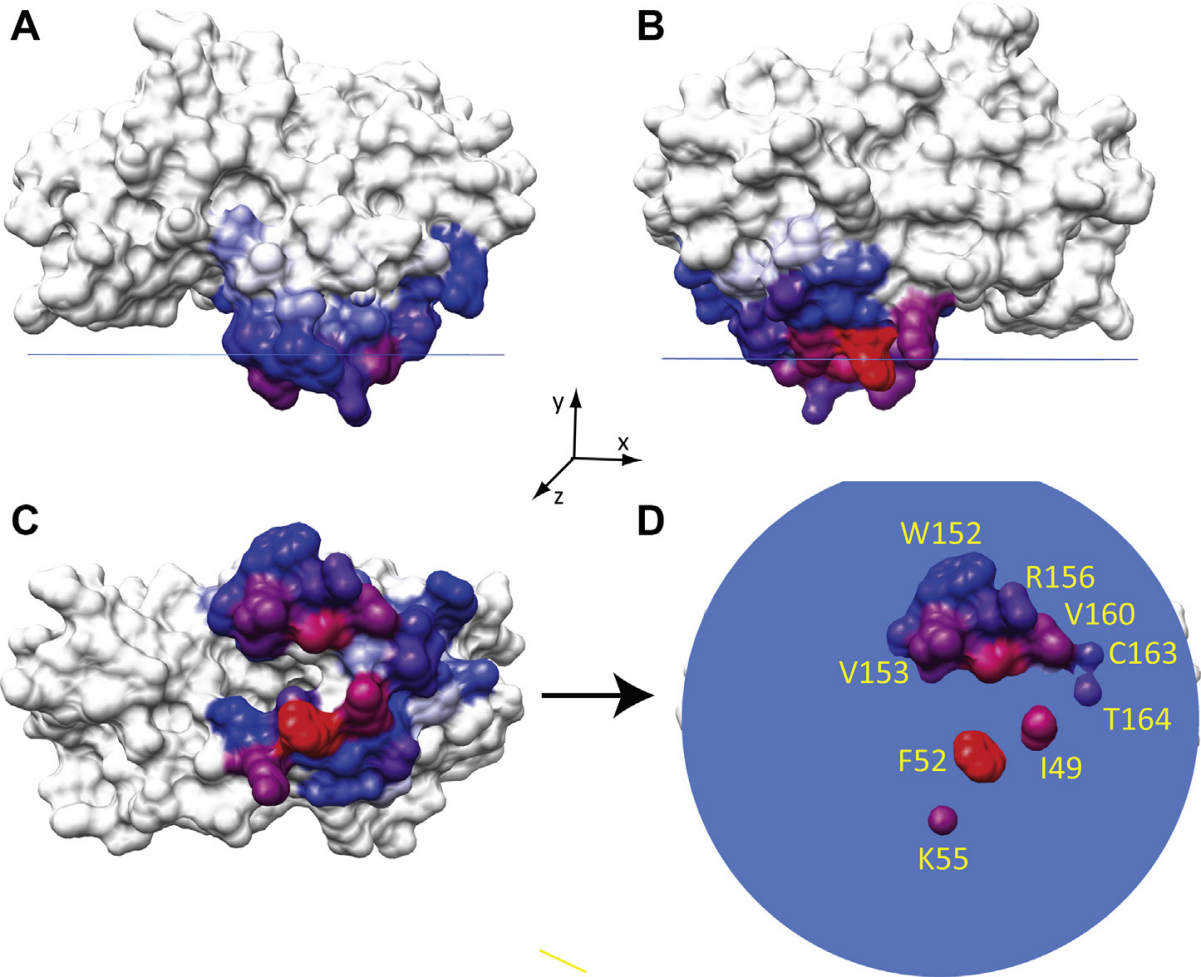
CPTP orientation during contact with a POPC membrane interface. A: CPTP (space-filling); (B) CPTP (space-filling); view = 180° rotation in the *y*-axis relative to (A). C: CPTP (space-filling); view = 90° rotation in the *x*-axis relative to (A). D: CPTP (space-filling); same view as (C) but with membrane polar/nonpolar interface (blue circle) as shown. The blue-to-red colors in CPTP correspond to membrane contact frequencies over the range of 54–88% (Table 1).

We next identified CPTP residues that most frequently maintained contact with the POPC bilayer during the 300 ns MD simulation. Table 1 shows the residues of the CPTP–C1P complex with membrane contact frequencies of 40% or higher. Notably, these residues reside mostly in α-helix 6 and in the N-terminal end of α-helix 2, are located on the external surface of CPTP, and are known to interact favorably with lipid bilayers. Figure 2A–D illustrate the positioning of many of the involved residues in relation to the POPC bilayer.

**Table 1:** POPC bilayer membrane contact frequencies of CPTP residues

Residue^a^	Location^b^	Contact Frequency^c^
PHE-52	α2-helix	87.86
ILE-49	α2-helix	73.45
LYS-55	α2-helix	69.78
ILE-53	α2-helix	61.04
ASP-56	α2-helix	51.23
THR-48	α2-helix	47.70
SER-51	α2-helix	46.56
ALA-157	α6-helix	76.05
VAL-160	α6-helix	69.11
VAL-153	α6-helix	67.71
VAL-154	α6-helix	65.98
ARG-156	α6-helix	60.37
THR-164	α6-helix	59.24
CYS-163	α6-helix	56.77
PRO-151	α6-helix	56.37
TRP-152	α6-helix	54.17
THR-159	α6-helix	41.09
SER-135	α5-helix	44.03
GLY-47	α1-α2 loop	61.51
ARG-133	α4-α5 loop	52.30
LEU-165	α6-α7 loop	43.76

a Data is for CPTP–C16-C1P complex.

b Location = CPTP PDB: 4k84.

c Values = % time interacting with POPC bilayer; CPTP residues listed have contact values >40%.

**Fig. 3:**
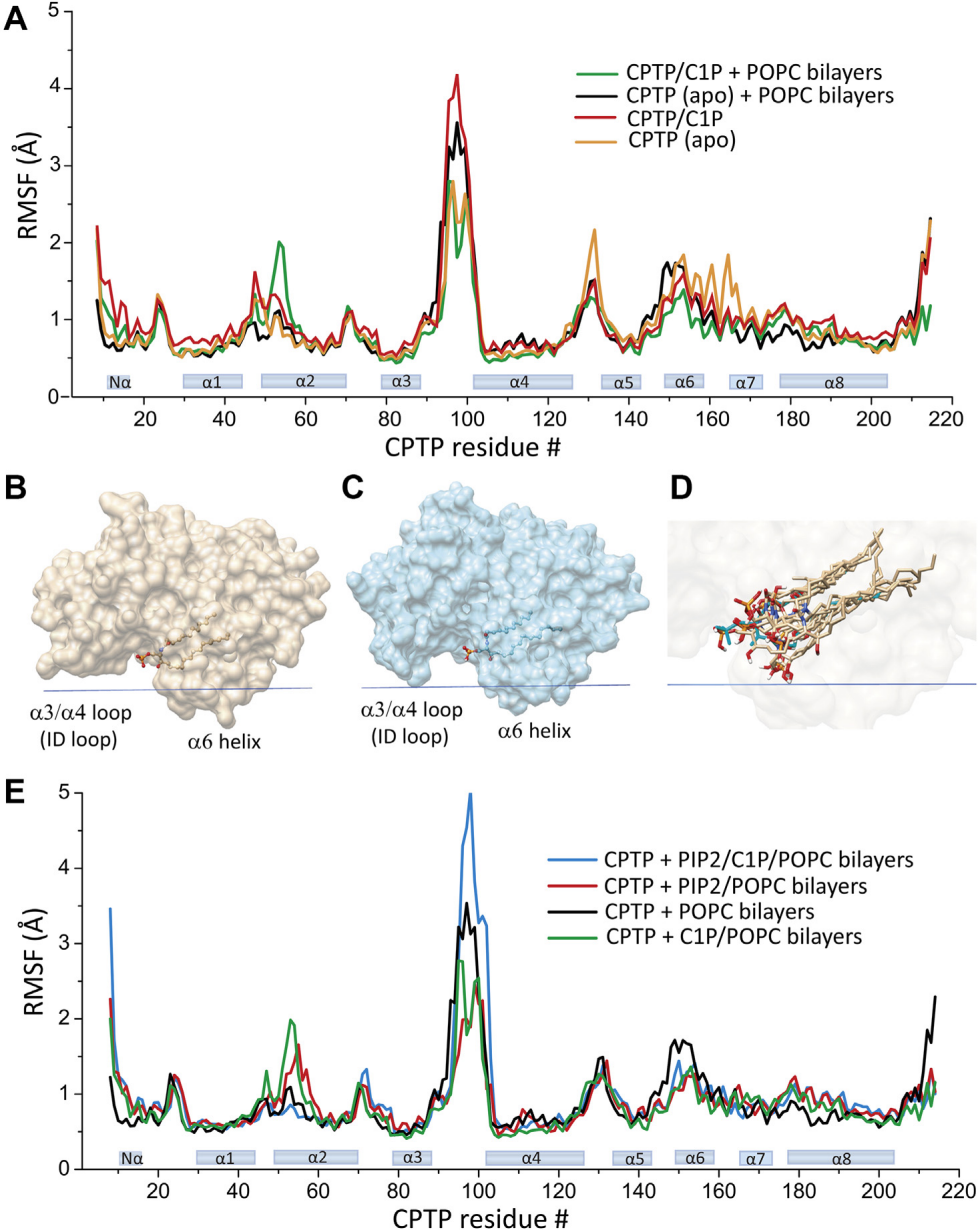
Dynamics of CPTP residues and C1P detected by MD simulation. A: RMSF plot of CPTP residues by sequence from the trajectories of 300 ns. Four different states associated with CPTP transfer of C1P are compared. B: Space-filling representation of CPTP/C1P crystal structure (PDB: 4k84). C: Snapshot of the CPTP–C1P complex from a cluster of the MD simulations indicates flexibility of α3/α4 helices connecting loop (ID loop) that enables close approach to the membrane lipid polar headgroups. D: Ten representative structures of C1P from 10 simulation clusters superpositioned to show the dynamics of C1P during simulation. C1P with cyan hydrocarbon chains is from the crystal structure (PDB: 4k84). E: RMSF plot of CPTP residues by sequence from 300 ns trajectories showing apo-CPTP interacting with bilayers composed of 100% POPC or POPC containing either C16-C1P or PI-(4,5)P_2_ or POPC containing both C16-C1P and PI-(4,5)P_2._

To obtain additional insights into how the local dynamics of various CPTP structural elements are affected by C1P binding to CPTP in the presence or absence of the POPC bilayer, we analyzed CPTP residue dynamics as a function of sequence (Fig. 3A). Perhaps not surprisingly, residues forming the connecting loops that link various helices tended to show increased dynamics compared with residues forming the α-helices. It is noteworthy that residues of the α-3/α-4 helices connecting loop exhibited exceptionally high overall dynamics that were clearly impacted by the presence or absence of the POPC bilayer and by the presence or absence of bound C1P. It also is noteworthy that the simultaneous presence of bound C1P and the POPC membrane increased the α-2 helix amino-end dynamics in CPTP but decreased the dynamics of the α-3/α-4 helices connecting loop and the COOH-terminal region residues. In apo-CPTP, in the absence of the POPC bilayer, relatively high dynamics were observed for not only the residues of the α-3/α-4 helices connecting loop, but also for residues of the α-4/α-5 helices connecting loop and many of those comprising the α-6 helix, the adjacent α-6/α-7 connecting loop, and the COOH-terminal region.

The α-3/α-4 helices connecting loop in the GLTP-fold has previously been implicated in the regulation of the differing glycolipid specificity in FAPP2-GLTPH domain and GLTP (12), and more recently has been found to harbor a PI-(4,5)P_2_ docking site in CPTP (21). Therefore, we analyzed the dynamics associated with the α-3/α-4 helices connecting loop in more detail. To do so, we partitioned the 1,500 total frames generated during the 300 ns MD simulation into 10 clusters using TTClust (41). TTClust helps ascertain which differences are only fluctuations of the system versus which are linked to different conformations. Figure 3B, C illustrate differing conformations that the α-3/α-4 helices connecting loop can assume when CPTP-containing bound C1P interacts with the POPC bilayer, as revealed by TTClust, relative to the X-ray crystallographic structure of the holo-protein determined in the absence of the POPC bilayer. The dynamic flexibility of the α-3/α-4 helices connecting loop enables close approach to the phosphoglyceride headgroup region of the bilayer (supplemental Video S1), consistent with recent findings indicating the presence of a PI-(4,5)P_2_ docking site within the α-3/α-4 helices connecting loop (21).

### Dynamics associated with bound C1P in CPTP

We also investigated the dynamics of 16:0-C1P when bound within the C1P-specific hydrophobic pocket of the protein. The 16:0-C1P polar headgroup was found to remain relatively flexible when interacting with the sphingolipid headgroup recognition site located near the protein surface (Fig. 3D). In contrast, the 16:0-C1P hydrocarbon chains remain relatively rigid and constrained when enveloped within the hydrophobic pocket consistent with a ‘tight fit’ within a minimally expanded pocket. Altogether, C1P remained within the confines of the pocket but displayed limited, dynamic back-and-forth sliding action along the pocket’s long axis. It is noteworthy that the 16:0-C1P orientation within the SL binding pocket of CPTP, that is, hydrocarbon chains inserted deeper than the polar headgroup, is opposite that of various other LTPs (13, 14, 46, 47, 48, 49, 50) in which the lipid polar headgroup is buried deeper than the hydrocarbon chains.

### Changes in CPTP dynamics induced by changes in bilayer lipid composition

To gain insights into apo-CPTP interaction with bilayers composed of differing lipid compositions. MD simulations were performed using the following: *i*) 100% POPC; *ii*) POPC containing C16-C1P; *iii*) POPC containing PI-(4,5)P_2_; and *iv*) POPC containing both C16-C1P and PI-(4,5)P_2_ (Fig. 3E). When present, C16-C1P became bound within the SL binding site of CPTP during the 300 ns simulation. The low C16-C1P bilayer concentration (2.5 mol%) was used to mimic the physiological situation of C1P being transported by CPTP soon after synthesis by ceramide kinase located in the *trans*-Golgi. The data show that the largest increases in the RMSF values for CPTP residues were observed for the α-3/α-4 helices connecting loop when the POPC bilayer contained both C1P and PI-(4,5)P_2_ (Fig. 3E ,blue trace). This finding is consistent with a recent study showing CPTP transfer activity stimulation via a PI-(4,5)P_2_ headgroup interaction site containing a di-Arg motif in the α-3/α-4 helices connecting loop (21).

### Lipid rearrangement induced by α-helix 6 of CPTP

**Fig. 4:**
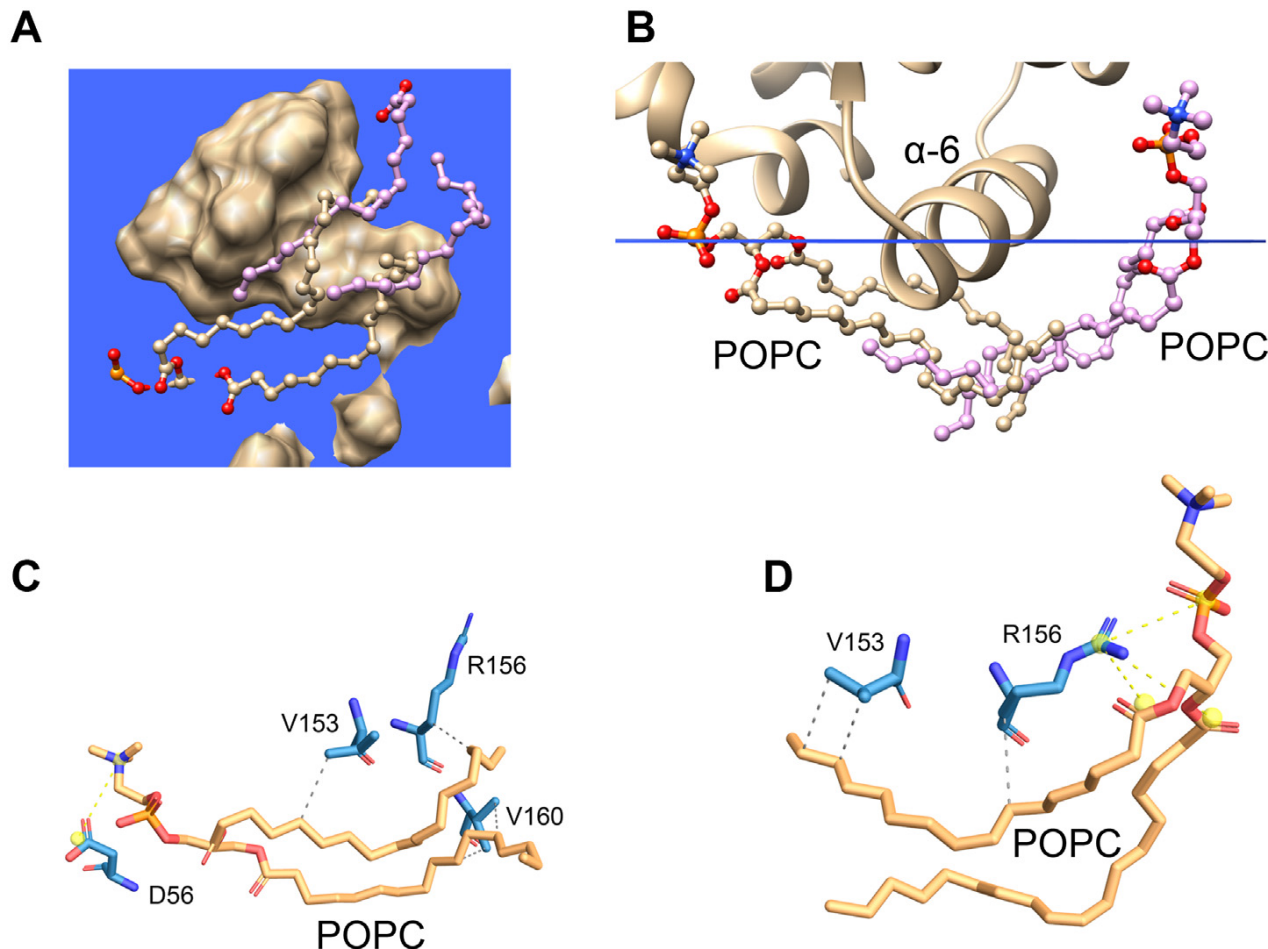
POPC rearrangement induced by CPTP α-6 helix detected by MD simulation. A: Membrane-penetrating region of CPTP (beige space-filling) viewed from inside the membrane looking toward the hydrocarbon/POPC polar headgroup interface (blue square). The POPC hydrophobic acyl chains (ball & stick format) become horizontally aligned with the membrane interface and ‘tunnel’ beneath the under-surface of CPTP by interacting with α-6 helix. B: Zoom view of α-6 helix region from end-on perspective. C: 3D map of interactions between CPTP α-6 helix residues (V153, R156, V160, and D56) and a POPC. D: 3D map of interactions between CPTP α-6 helix residues (V153 and R156) and a POPC.

**Fig. 5:**
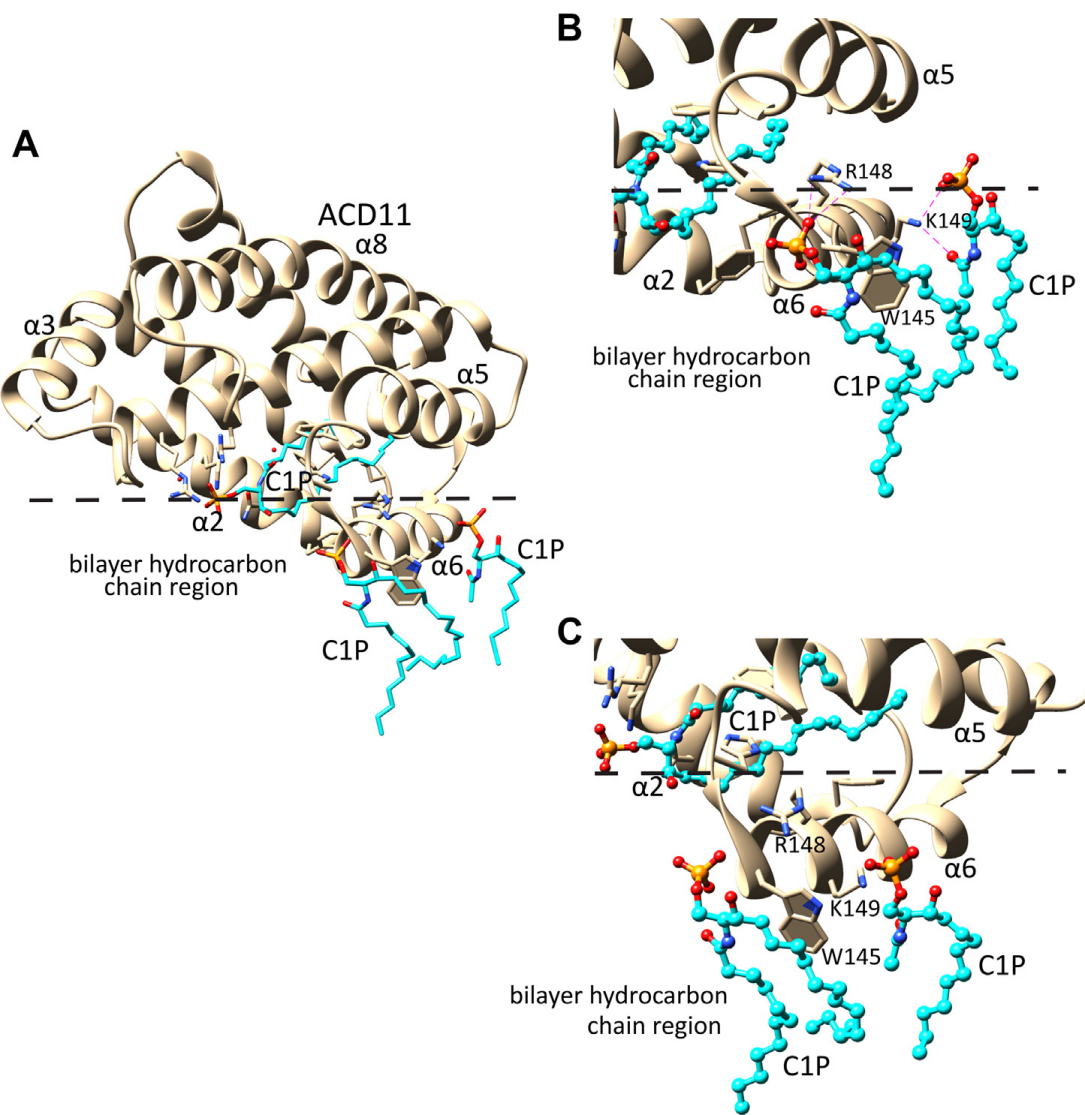
C1P interaction with α-6 helix detected by X-ray crystallography of ACD11/C1P. Two C1P interaction sites are evident on α-6 helix other than C1P occupying the hydrophobic binding pocket of CPTP. The dashed lines indicate the position of the polar-to-nonpolar interface as defined by the acyl ester linkages of POPC. C1P molecules interacting with the α-6 helix and the hydrophobic binding pocket are depicted with cyan hydrocarbon chains, red oxygens, blue nitrogens, and orange phosphates. A: Structure of ACD11/C1P (PDB: 4NTI). B, C: Zoom views from end-on and parallel perspectives of α-6 helix showing the positioning of C1P-interacting residues R148, K149, and W152.

We next investigated the impact of CPTP on the dynamic conformational states of nearby POPC molecules within the lipid-bilayer matrix. Notably, α-helix 6 was found to be unique in its ability to strongly affect POPC dynamic conformations, with respect to the lipid’s orientation within the bilayer. As illustrated in Fig. 4, the POPC hydrocarbon chains were found to partially ‘wrap’ around the α-helix 6 membrane-facing exterior surface, whereas R156 interacted with the POPC phosphate and acyl-amide polar groups (Fig. 4D). This observation led us to hypothesize that the bilayer normal positioning of α-helix 6 combined with its preponderance of nonpolar residues and other residues (e.g., Arg and/or Lys) that are able to favorably interact with phosphoglyceride amphiphiles promote the large conformational changes observed in the interacting POPC hydrocarbon chains. We further hypothesized that the ability of the α-6 helix exterior surface to induce repositioning and partial ‘wrapping’ of the POPC hydrocarbon chains could reflect a mechanism used to orient and guide C1P to and from the nearby C1P-selective binding pocket. To gain further insights, we first examined X-ray crystallographic diffraction data showing the different ways that C1P can interact with the plant CPTP homolog, ACD11 (PDB: 4NTI) (11). In ACD11/C1P, cocrystallization of protein and sphingolipid reveals C1P interacting with α-6 helix as well as occupying the C1P-specific binding/transfer site (Fig. 5A). Close examination of the α-6 helix interactions with C1P shows R148-K149 di-cationic site involvement that includes formation of a salt bridge by K149 with the C1P-phosphate group and hydrogen bonding by the K149 side chain with the carbonyl oxygen of the amide linkage in a second C1P molecule (Fig. 5B). Also evident is the R148 hydrogen bonding with the phosphate group of a C1P molecule, whereas its hydrocarbon chains undergo van der Waals contacts with the indole ring of W145 in ACD11 (Fig. 5C).

**Fig. 6:**
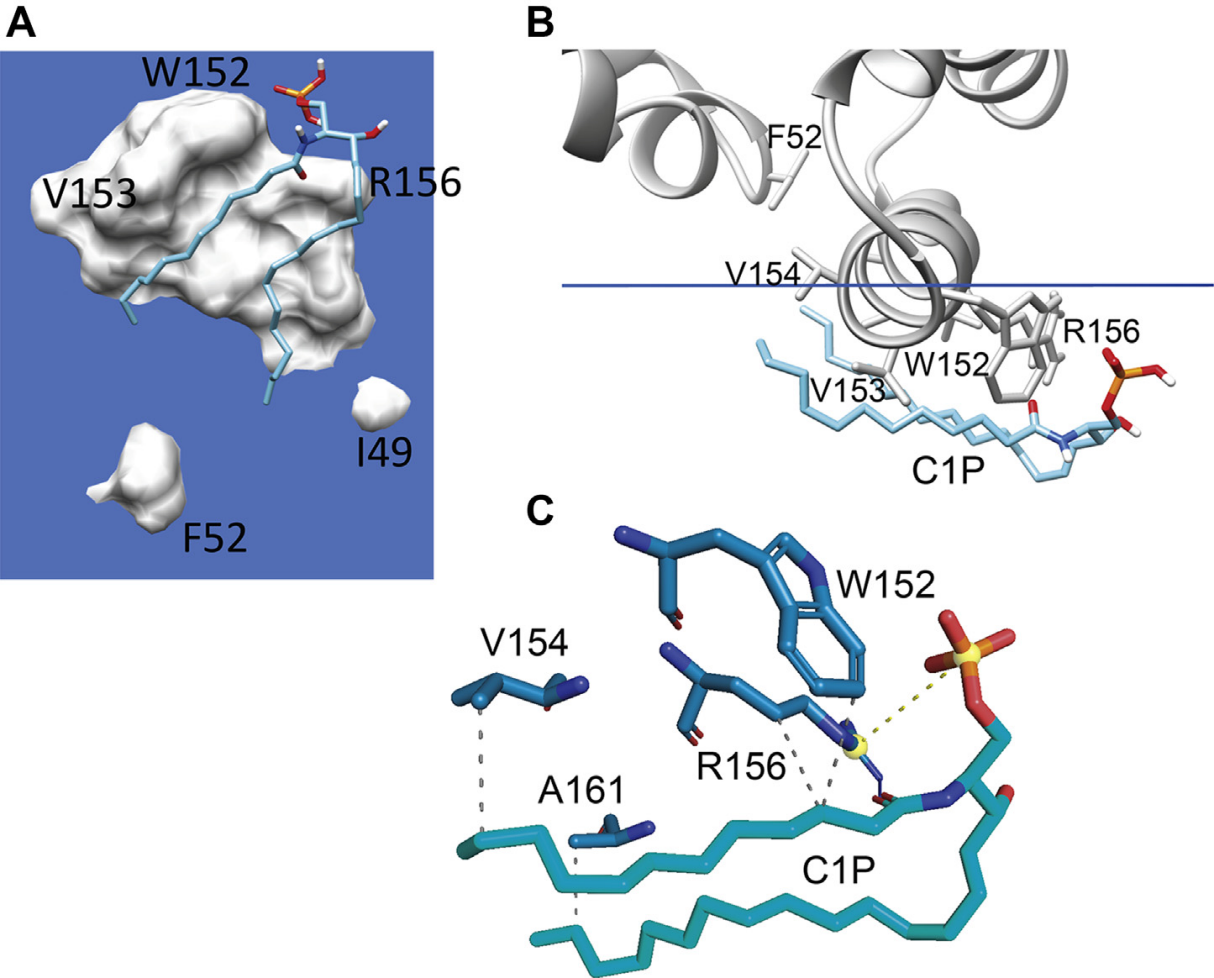
C16-C1P rearrangement induced by CPTP α-6 helix during MD simulation (e.g., supplemental Video S2). C16-C1P is shown in stick representation with cyan hydrocarbon chains, red oxygen, blue nitrogen, and orange phosphate. A: View from beneath the bilayer interface (blue). CPTP residues are shown as space-filling. B: End-on view of α-6 helix aligned with the bilayer interface (blue line) showing the repositioning of C1P induced by interacting α-6 helix residues R156, W152, V153, and V154. C: Zoom view of C1P interacting with α-6 helix residues W152, R156, V154, and A161.

Because of the existence of the structurally analogous R155-R156 di-cationic site in CPTP (12), we used MD simulations to test whether the ACD11 crystallographic findings might reflect a role for α-helix 6 R156 in the uptake of C1P. To do so, we initially generated a model of a POPC bilayer containing one C1P molecule positioned such that the C1P headgroup was near the α-6 helix di-Arg group of apo-CPTP. The orientation and penetration depth of the apo-CPTP into the lipid bilayer relied on OPM/PPM modeling followed by placement of the CPTP/C1P/POPC membrane system in a water box using the Charmm-GUI server. MD simulations were then performed for 20 ns on the final system to determine if the dynamic C16-C1P conformation is affected by CPTP in similar ways as observed for POPC. Figure 6 shows that C1P did transition to a more membrane-interface-parallel orientation with its hydrocarbon chains interacting with the membrane-exposed surface of α-6 helix in CPTP (supplemental Video S2). The contact frequencies of various residues in CPTP α-6 helix with C16-C1P during first 20 ns of the MD simulation are summarized in Table 2. Interestingly, the high contact frequencies for Trp152 and Arg156 in the simulation model correspond well with the crystallographic close contacts observed with analogous, similarly positioned residues in plant CPTP ACD11. Note that when C16-C1P in the POPC membrane was replaced by C1P with a short hexanoyl acyl chain (6:0), the C6-C1P phosphate headgroup remained poorly engaged with α-6 helix (that contains the R155-R156 di-Arg motif) and failed to wrap beneath α-6 helix during the initial 20 ns of the MD simulation (supplemental Video S3), a finding that could reflect competition by the more abundant POPC, which also can interact with R156 and has two long acyl chains that can wrap around the membrane-facing external surface of α-6 helix.

**Table 2:** CPTP α-helix 6 residue initial contact frequency with C16-C1P in POPC bilayer

α6-Helix Residue^a^	Contact Frequency^b^
TRP-152	99%
VAL-153	55%
VAL-154	3%
ARG-156	97%
ALA-157	58%
VAL-160	24%
ALA-161	4%

a α-6-helix of CPTP.

b Values = % interaction time with C16-C1P versus POPC during first 20 ns of simulation.

## Discussion

The human branch of the GLTP superfamily consists of four members: GLTP, FAPP2, CPTP, and GLTPD2 (14). Orthologs of GLTP, FAPP2, and CPTP have also been identified in many other eukaryotes and characterized in filamentous fungi and plants (5, 11, 12, 14, 51, 52, 53, 54, 55, 56, 57). From the structural perspective, the most extensively characterized superfamily members are human GLTP and CPTP, and the plant CPTP ortholog, ACD11, which account for 37 X-ray crystallographic deposits (90%) in the Protein Data Bank. The MD simulations presented here provide the first insights into the conformational dynamics of CPTP transition states linked to the sphingolipid transfer process such as CPTP interaction with the membrane bilayer and the binding of the C1P cargo sphingolipid by CPTP. No such insights have existed previously for any GLTP superfamily member.

The challenges associated with various modeling options involving membrane interaction by lipid transfer proteins and other peripheral membrane proteins have recently been reviewed by (58). Extensive efforts for the lipid parameterization set have led to widespread use of CHARMM36, which can be applied without the need for an external surface tension while faithfully reproducing the physicochemical properties of a wide variety of lipid bilayers (59, 60). Our choice for using POPC for initial MD simulation studies of CPTP conformational dynamics associated with fluid-phase bilayer matrix interaction reflects this PC species often being a major biomembrane component as well as having an asymmetric acyl composition with the *sn*-1 chain being long and saturated (C ≥ 16) and the long *sn*-2 chain (C ≥ 18) containing *cis* unsaturation. Such an acyl chain arrangement has interesting biological consequences including more efficient membrane vesiculation and lower membrane permeability compared with phosphoglycerides containing two saturated or polyunsaturated chains (61). As mentioned in the Materials and methods, we performed our MD simulations using POPC at average surface areas of ∼68.6 Å^2^/molecule as a means to mimic the lipid packing in planar fluid-phase PC membranes with *sn*-2 monounsaturated acyl chains. Future MD simulation studies will be needed to address the effect of changes in average POPC surface area/molecule on CPTP interaction as associated with curvature-stressed outer surfaces present in small lipid bilayer vesicles often used for protein-mediated lipid intervesicular transfer measurements due to their high response sensitivity, that is, faster lipid transfer rates. It is noteworthy that previous lipid monolayer studies involving GLTP (44, 45, 62, 63) have shown that surface pressure changes (that alter the average surface area/lipid molecule) do affect the GLTP-mediated glycolipid transfer rate and GLTP penetration into lipid monolayers composed of various phosphoglycerides. Yet, the air/water interface of lipid monolayers present a different environment for the lipid-acyl chains than that of the bilayer mid-plane, complicating direct comparisons between POPC monolayers versus POPC bilayers based on lipid packing. In any case, we expect that future MD simulations will provide bilayer data that will be qualitatively supported by the earlier lipid monolayer findings for GLTP, while also providing insights into the CPTP regions most impacted with respect to their conformational dynamics.

MD simulations of other lipid binding/transfer proteins include studies of various fatty acid binding proteins, plant nonspecific lipid transfer proteins, and phosphatidylinositol transfer protein (64, 65, 66, 67, 68, 69, 70, 71). The fundamentally different structural organizations of the preceding proteins, sometimes including stabilizing internal disulfides, prevent meaningful insights from being gained into the conformational dynamics associated by comparisons with the structurally unique all-α-helical GLTP-fold of CPTP. In the GLTP-fold, intramolecular conformational stability and dynamics are maintained and regulated by cation-π and π- π stacking interactions, van der Waals contacts, and salt bridges, but not by disulfide bridging (1, 9, 12, 54). Accordingly, thermally-induced denaturing profiles for GLTP superfamily members such as GLTP indicate rather low unfolding temperatures (≤55°C) (5, 9, 72).

Our MD simulations took advantage of existing X-ray crystallographic data that define CPTP global conformation and of OPM/PPM modeling for initial protein positioning and orientation for membrane interaction. Not surprisingly, internally oriented residue side-chains directly involved in CPTP forming its GLTP-fold tend to exhibit less flexibility than residue side-chains residing on the CPTP outer surface. In addition, CPTP regions consisting of α-helices are generally less dynamic than loops linking the α-helices together. No large scale conformational changes associated with ‘transition’ states/stages directly related to CPTP function are evident in the dynamics exhibited by apo-CPTP and holo-CPTP in the absence and presence of the POPC bilayer. Yet, it is noteworthy that residues of the α-3/α-4 helices connecting loop collectively exhibit relatively high overall dynamics that clearly are impacted by the presence or absence of the POPC bilayer and by the presence or absence of bound C1P. Other CPTP regions measurably but less so affected by similar conditions include increased dynamics by α-helix 2 amino-end residues but decreased dynamics by the α-3/α-4 helices connecting loop and the COOH-terminal region residues. The findings are consistent with these protein regions being located in close proximity to the membrane bilayer interface and near the sphingolipid recognition center where they likely play a role in C1P acquisition/release during the SL intermembrane-transfer process. The finding of the α-3/α-4 helices connecting loop dynamically approaching the membrane surface is noteworthy because recent studies have implicated this loop in harboring a PI-(4,5)P_2_ docking site that enhances the C1P transfer activity of CPTP (21) and regulating the differing glycolipid specificity in FAPP2-GLTPH domain and GLTP (12).

**Fig. 7:**
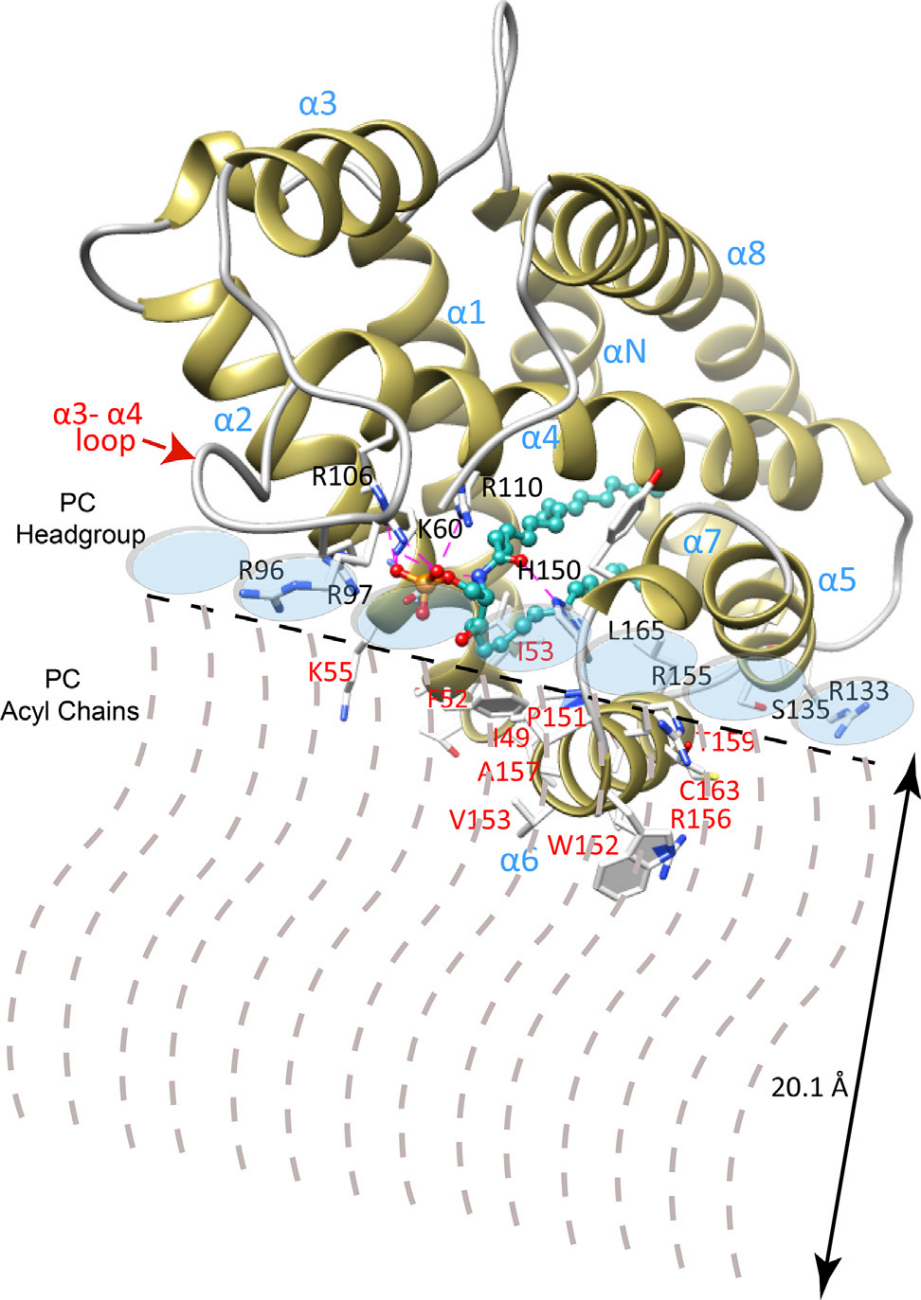
Scale model of CPTP orientation and penetration depth into POPC bilayer leaflet revealed by MD simulations. CPTP interaction is dominated by α-6 helix and the N-terminal region of α-2 helix. Residues that penetrate into the upper hydrocarbon region are labeled in red. The relatively shallow penetration by the protein supports a model for CPTP scanning the membrane surface to acquire C1P via a lipid-chain reorienting mechanism involving α-6 helix, as illustrated by Fig. 8.

The MD simulations here indicate that α-helix 6 of CPTP is the dominant structural element driving membrane bilayer interaction, a finding supported by earlier mutation and OPM/PPM modeling studies of the GLTP-fold of human GLTP (1, 4, 5, 10, 20, 22, 43, 44, 45, 46, 47, 48, 49). Indeed, GLTP interaction with the POPC bilayer has previously been found to be relatively nonperturbing, as expected for shallow penetration by α-6 helix that is limited to one side of the bilayer (43). The MD simulations also support the overall position and orientation of CPTP remaining relatively stable during interaction with the POPC bilayer, although penetration depths for α-helix 6 fluctuate modestly with respect to the average location of surrounding lipid-bilayer interface. Our data enable estimation of penetration depths by CPTP structural elements that directly interact with the POPC bilayer, that is α-helix 6, the N-terminal region of α-2 helix, α-3/α-4 helices connecting loop. To do so, we initially considered structural parameters determined for liquid-crystalline (Lα phase) bilayers consisting of di-oleoyl PC (35, 73). Such parameters include the distances of choline (21.87 Å), phosphate (20.17 Å), and the acyl chain carbonyl groups (15.98 Å) from the bilayer mid-plane as well as phosphorylcholine headgroup parameters such as orientation (22 ± 4°) respect to the bilayer interface and the nitrogen-to-phosphate distance (4.5 Å). Using the acyl carbonyl groups as markers of the headgroup/hydrocarbon boundary (26, 27, 28), the polar headgroup region thickness equals ∼8.5–9.0 Å (based on ∼5.9 Å for the acyl carbonyl group-to-choline nitrogen distance allowing plus 2.5–3.0 Å for hydration of choline). Notably, in the CPTP/POPC membrane, MD simulation yields average distances of 21.3 Å for choline-to-bilayer mid-plane and 20.1 Å for phosphate-to-bilayer mid-plane, values that agree well with the data obtained by X-ray and neutron diffraction (35, 73). Based on this agreement, we estimated the MD simulation distances from the bilayer mid-plane to the α-helix 6 Trp152 and Arg156 side-chains to be 14.6 (±1.3) and 15.3 (±1.0) Å, respectively. Table 3 summarizes this data as well as distances calculated for residues in the N-terminal region of α-helix 2 (e.g., Ile49, Phe52, Ile53, and Lys55) that embed in the POPC bilayer and residues of the α-3/α-4 helices connecting loop (R^90^LVDLERRSHHPE^102^) that dynamically approach the POPC headgroups of the bilayer. Figure 7 provides a summary schematic (drawn to scale) of CPTP interacting with one half of a POPC bilayer.

**Table 3:** Model for CPTP/C16-C1P penetration depth into POPC bilayer

Residue^a^	Location^b^	Distance from Bilayer Mid-Plane (Å)^c^
TRP-152	α-helix 6	14.6 (±1.3)
VAL-153	α-helix 6	10.4 (±1.4)
VAL-154	α-helix 6	15.1 (±1.1)
ARG-156	α-helix 6	15.3 (±1.0)
ALA-157	α-helix 6	14.5 (±1.0)
VAL-160	α-helix 6	14.3 (±0.9)
PHE-52	α-helix 2	16.5 (±1.6)
ILE-49	α-helix 2	17.7 (±0.9)
LYS-55	α-helix 2	17.2 (±1.3)
ILE-53	α-helix 2	18.2 (±2.0)
GLU-95	α 3/α 4 loop	27.2 (±2.8)
ARG-96	α-3/α-4 loop	22.7 (±2.7)
ARG-97	α-3/α-4 loop	22.6 (±1.8)
SER-98	α-3/α-4 loop	22.3 (±1.9)
HIS-99	α-3/α-4 loop	20.1 (±2.5)
HIS-100	α-3/α-4 loop	24.4 (±2.6)

a Data is for CPTP–C16-C1P complex.

b Location = CPTP (PDB: 4k84).

c In POPC bilayer, phosphate distance from bilayer mid-plane ≈20.1 Å (35, 73).

**Fig. 8:**
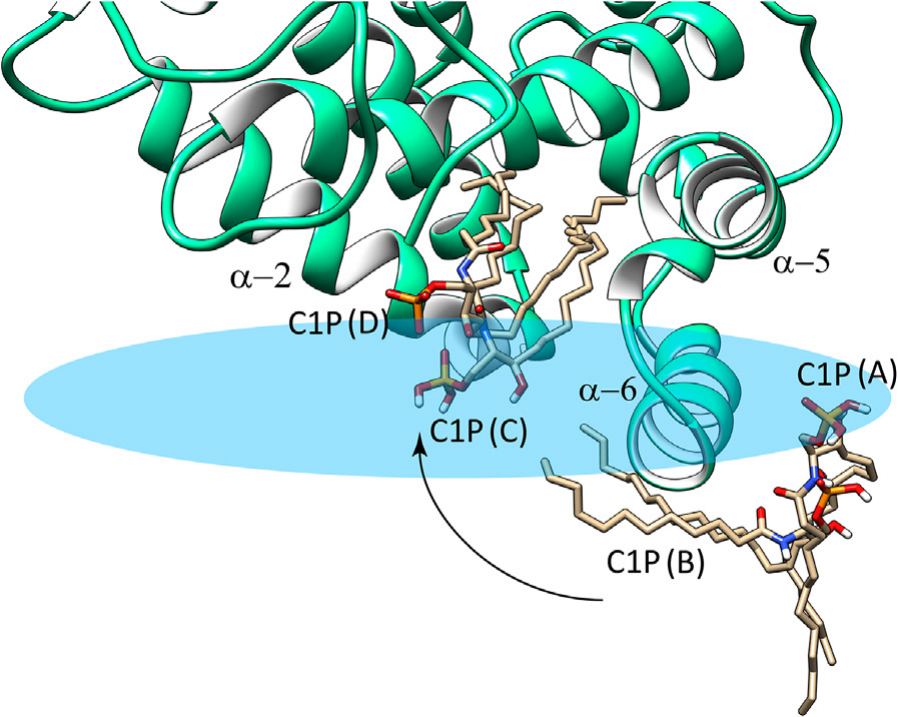
A model illustrating the functional role of α-6 helix for reorienting and guiding C1P into the binding pocket of CPTP from membrane. The progression of events that lead to C1P binding is shown by four different C1P conformations. C1P (A) represents C1P positioned normally with the POPC bilayer; C1P (B) represents C1P that has become reoriented and repositioned by interaction with α-6 helix where the C1P hydrocarbon chains interact with Trp152 and Val153, whereas the CIP phosphate interacts with di-Arg motif (R155 and R156). The reoriented C1P becomes much more aligned with the bilayer interface thus enabling access to the hydrophobic binding pocket of CPTP. C1P (C) is shown sliding into the binding pocket of CPTP. C1P (D) represents C1P secured within the hydrophobic pocket due to the specific interactions of the C1P polar headgroup, that is, phosphate and acyl-amide linkage, with residues that define the C1P recognition motif as previously identified by X-ray crystallography and mutation analyses.

Notably, the MD simulations indicate that α-6 helix does more than function as a simple membrane docking element for CPTP. Compared with the other membrane contact regions of CPTP, α-6 helix exerts strong and unique effects on the dynamic conformations of POPC and C1P. When these lipids come into direct contact with α-6 helix via translational diffusion within the bilayer, their hydrocarbon chains transition from bilayer interface normal to bilayer interface aligned orientation. R156 and W152 located near the N-terminal end of α-6 helix are especially important for the reorientation process based on their nearly sustained dynamic contacts with the lipid. The finding for C1P is supported by X-ray crystallography structural data for plant CPTP, ACD-11 (PDB: 4NTI) showing that C1P can interact with the N-terminal end of α-6 helix N-end via K149, R148, and W145 (11). Notably, in control MD-simulations involving CPTP-R156L mutant (supplemental Video S4), the phosphate headgroup of C16-C1P did not engage strongly with the mutated α-6 helix, and the C16-C1P hydrocarbon chains did not wrap beneath α-6 helix as observed for wild type CPTP. However, this observation should not be construed as implying that R156 is absolutely specific for C1P uptake or essential for CPTP-mediated C1P transfer. Indeed, R156 also was observed interacting with the phosphate and acyl-amide groups of POPC (Fig. 4D). Moreover, we know from previous studies of GLTP-fold proteins (including CPTP) that various other α-6 helix residues contribute to the membrane engagement needed for sphingolipid intervesicular transfer to occur. In the case of CPTP, such residues include Trp, Val, Ile, Phe, along with di-Arg (10). In the case of the R155/R156 di-Arg motif, CPTP-mediated C1P transfer has been partially addressed by R155 point mutation (CPTP-R155Q) in which slowing, but not complete inhibition, of C1P transfer was observed and decreased CPTP membrane association (21). A more comprehensive study will be needed to further clarify potential ‘dual role’ of the di-Arg region in the α-6 helix in both CPTP membrane association and C1P transfer. Taken together, these findings lead us to propose that the N-end of α-6 helix functions to reorient and guide C1P to the CPTP binding pocket region. The C1P dynamic behavior that supports our hypothesis for α-6 helix involvement in C1P uptake by CPTP is summarized in Fig. 8. Initially, the C1P (A) molecule is aligned with the POPC molecules forming one lamellar surface of the bilayer. Interaction of the C1P phosphate headgroup with the R155/R156 di-Arg region in α-6 helix serves to help trigger dynamic reorientation that promotes interaction of the C1P nonpolar hydrocarbon chains with α-6 helix Trp152 and Val153 via van der Waals contacts, resulting in a horizontal conformation with respect to the bilayer normal (Fig. 8, C1P (B)). The horizontally-reoriented C1P hydrocarbons are more favorably positioned for gaining entry into the C1P-specific binding pocket. This dynamic repositioning, coupled with guidance by Phe52, Ile49, and Ile53 at the N-terminal region of α-2 helix, facilitates C1P hydrocarbon chain ‘tunneling’ into the C1P binding pocket that is lined with hydrophobic residues (Fig. 8 by C1P (C)). Selectivity for C1P can then occur via the sphingolipid headgroup recognition center interaction with the C1P phosphate and acyl-amide linkage via a conserved Asp^56^-Lys^60^_∗∗∗∗_Arg^106^-Arg^110^_∗∗∗∗_His^150^ three-dimensional motif (5, 10, 12, 14). The final bound C1P position in the CPTP binding pocket is illustrated in Fig. 8 (C1P (D)).

Although the ability of α-6 helix to deform and reorient the hydrocarbon chains of C16-C1P is clear, our present data do not reveal whether the C1P acyl chain enters the CPTP hydrophobic pocket before the sphingosine chain. This kind of oriented C1P entry into the CPTP hydrophobic pocket has been implied from X-ray crystallography data that sometimes capture a ‘sphingosine-out’ C1P binding mode that may play a role in regulating transient homo-dimerization on the membrane surface (5, 6, 10). Future experiments will be needed to address the preceding issue including whether the C1P acyl chain initially undergoes more frequent deformations than the sphingosine chain due to an oriented C1P interaction with α-6 helix. What currently is clear is that the CPTP binding pocket samples various membrane lipids in sorting-like fashion but can only fully engage with C1P due to the specific C1P recognition interactions. It is interesting to speculate that the dynamic conformational changes to the CPTP α-3/α-4 helices connecting loop that occur when both C1P and PI-(4,5)P_2_ are present may be offset or altered when CPTP/C1P encounters a membrane surface containing other ‘regulator’ phosphoglycerides such as phosphatidylserine or phosphatidic acid to facilitate C1P release (20). Future experiments will be needed to further test this hypothesis.

## Conclusions

The findings gained in the present study from the MD simulations provide the first insights into the conformational dynamics that enables human CPTP to acquire and deliver C1P during interaction with membranes. Penetration by CPTP into the membrane bilayer is confined mostly to α-6 helix and the N-terminal end of α-2 helix, involves only one bilayer leaflet and is relatively shallow. Dynamic, large-scale conformational changes for CPTP during membrane interaction or C1P uptake are minimal except for the α-3/α-4 helices connecting loop which recently was shown to interact with certain membrane-embedded phosphoinositides. Besides functioning as a shallow membrane-anchoring element, the ability of α-6 helix to deform and reorient membrane lipids is consistent with a proposed functional role in C1P uptake. This finding is noteworthy because the lipid orientations within the binding pockets of various other lipid binding/transfer proteins support a ‘polar headgroup first’ entry via a different mechanism rather than the ‘hydrocarbon chain first’ entry that is proposed for the GLTP-fold. It is interesting to note that integral membrane channel proteins that function in the transbilayer migration of specific lipids contain α-helices with similar features that aid in the lipid reorientation needed for successful translocation across the membrane (74, 75, 76). Finally, gaining insights into CPTP is important because of this protein’s recently revealed role in regulating cytosolic phospholipase A_2_α action that drives pro-inflammatory eicosanoid production as well as in autophagy-dependent inflammasome assembly needed for production and release of interleukins (IL-1β and IL-18) by surveillance cells and induction of pyroptosis.

## Data availability

All data pertaining to the article and the supporting information are available upon request by contacting Yong-Guang Gao <gaoxx110@umn.edu> or Rhoderick E. Brown <reb@umn.edu>.

## Supplemental data

This article contains supplemental data.

## Conflict of interest

The authors declare no competing interests.

## References

[1] Malinina L., Malakhova M.L., Teplov A., Brown R.E., Patel D.J. (2004). Structural basis for glycosphingolipid transfer specificity. Nature.

[2] Malinina L., Malakhova M.L., Kanack A.T., Lu M., Abagyan R., Brown R.E., Patel D.J. (2006). The liganding of glycolipid transfer protein is controlled by glycolipid acyl structure. PLoS Biol..

[3] Airenne T.T., Kidron H., Nymalm Y., Nylund M., West G., Mattjus P., Salminen T.A. (2006). Structural evidence for adaptive ligand binding of glycolipid transfer protein. J. Mol. Biol..

[4] Brown R.E., Mattjus P. (2007). Glycolipid transfer proteins. Biochim. Biophys. Acta.

[5] Malinina L., Simanshu D.K., Zhai X., Samygina V.R., Kamlekar R., Kenoth R., Ochoa-Lizarralde B., Malakhova M.L., Molotkovsky J.G., Patel D.J., Brown R.E. (2015). Sphingolipid transfer proteins defined by the GLTP-fold. Q. Rev. Biophys..

[6] Malinina L., Patel D.J., Brown R.E. (2017). How alpha-helical motifs form functionally diverse lipid-binding compartments. Annu. Rev. Biochem..

[7] Samygina V.R., Popov A.N., Cabo-Bilbao A., Ochoa-Lizarralde B., Goni-de-Cerio F., Zhai X., Molotkovsky J.G., Patel D.J., Brown R.E., Malinina L. (2011). Enhanced selectivity for sulfatide by engineered human glycolipid transfer protein. Structure.

[8] Samygina V.R., Ochoa-Lizarralde B., Popov A.N., Cabo-Bilbao A., Goni-de-Cerio F., Molotkovsky J.G., Patel D.J., Brown R.E., Malinina L. (2013). Structural insights into lipid-dependent reversible dimerization of human GLTP. Acta Crystallogr. D Biol. Crystallogr..

[9] Kamlekar R.K., Simanshu D.K., Gao Y.G., Kenoth R., Pike H.M., Prendergast F.G., Malinina L., Molotkovsky J.G., Venyaminov S.Y., Patel D.J., Brown R.E. (2013). The glycolipid transfer protein (GLTP) domain of phosphoinositol 4-phosphate adaptor protein-2 (FAPP2): structure drives preference for simple neutral glycosphingolipids. Biochim. Biophys. Acta.

[10] Simanshu D.K., Kamlekar R.K., Wijesinghe D.S., Zou X., Zhai X., Mishra S.K., Molotkovsky J.G., Malinina L., Hinchcliffe E.H., Chalfant C.E., Brown R.E., Patel D.J. (2013). Non-vesicular trafficking by a ceramide-1-phosphate transfer protein regulates eicosanoids. Nature.

[11] Simanshu D.K., Zhai X., Munch D., Hofius D., Markham J.E., Bielawski J., Bielawska A., Malinina L., Molotkovsky J.G., Mundy J.W., Patel D.J., Brown R.E. (2014). Arabidopsis accelerated cell death 11, ACD11, is a ceramide-1-phosphate transfer protein and intermediary regulator of phytoceramide levels. Cell Rep..

[12] Ochoa-Lizarralde B., Gao Y.G., Popov A.N., Samygina V.R., Zhai X., Mishra S.K., Boldyrev I.A., Molotkovsky J.G., Simanshu D.K., Patel D.J., Brown R.E., Malinina L. (2018). Structural analyses of 4-phosphate adaptor protein 2 yield mechanistic insights into sphingolipid recognition by the glycolipid transfer protein family. J. Biol. Chem..

[13] Mattjus P. (2016). Specificity of the mammalian glycolipid transfer proteins. Chem. Phys. Lipids.

[14] Mishra S.K., Gao Y.G., Zou X., Stephenson D.J., Malinina L., Hinchcliffe E.H., Chalfant C.E., Brown R.E. (2020). Emerging roles for human glycolipid transfer protein superfamily members in the regulation of autophagy, inflammation, and cell death. Prog. Lipid Res..

[15] D'Angelo G., Polishchuk E., Di Tullio G., Santoro M., Di Campli A., Godi A., West G., Bielawski J., Chuang C.C., van der Spoel A.C., Platt F.M., Hannun Y.A., Polishchuk R., Mattjus P., De Matteis M.A. (2007). Glycosphingolipid synthesis requires FAPP2 transfer of glucosylceramide. Nature.

[16] D'Angelo G., Uemura T., Chuang C.C., Polishchuk E., Santoro M., Ohvo-Rekila H., Sato T., Di Tullio G., Varriale A., D'Auria S., Daniele T., Capuani F., Johannes L., Mattjus P., Monti M. (2013). Vesicular and non-vesicular transport feed distinct glycosylation pathways in the Golgi. Nature.

[17] Halter D., Neumann S., van Dijk S.M., Wolthoorn J., de Maziere A.M., Vieira O.V., Mattjus P., Klumperman J., van Meer G., Sprong H. (2007). Pre- and post-Golgi translocation of glucosylceramide in glycosphingolipid synthesis. J. Cell Biol..

[18] Yamaji T., Hanada K. (2015). Sphingolipid metabolism and interorganellar transport: localization of sphingolipid enzymes and lipid transfer proteins. Traffic.

[19] Kumagai K., Hanada K. (2019). Structure, functions and regulation of CERT, a lipid-transfer protein for the delivery of ceramide at the ER-Golgi membrane contact sites. FEBS Lett..

[20] Zhai X., Gao Y.G., Mishra S.K., Simanshu D.K., Boldyrev I.A., Benson L.M., Bergen H.R., Malinina L., Mundy J., Molotkovsky J.G., Patel D.J., Brown R.E. (2017). Phosphatidylserine stimulates ceramide 1-phosphate (C1P) intermembrane transfer by C1P transfer proteins. J. Biol. Chem..

[21] Gao Y.G., Zhai X., Boldyrev I.A., Molotkovsky J.G., Patel D.J., Malinina L., Brown R.E. (2021). Ceramide-1-phosphate transfer protein (CPTP) regulation by phosphoinositides. J. Biol. Chem..

[22] Mishra S.K., Gao Y.G., Deng Y., Chalfant C.E., Hinchcliffe E.H., Brown R.E. (2018). CPTP: a sphingolipid transfer protein that regulates autophagy and inflammasome activation. Autophagy.

[23] Mishra S.K., Stephenson D.J., Chalfant C.E., Brown R.E. (2019). Upregulation of human glycolipid transfer protein (GLTP) induces necroptosis in colon carcinoma cells. Biochim. Biophys. Acta Mol. Cell Biol. Lipids.

[24] Yang F., Guan Y., Feng X., Rolfs A., Schluter H., Luo J. (2019). Proteomics of the corpus callosum to identify novel factors involved in hypomyelinated Niemann-Pick Type C disease mice. Mol. Brain.

[25] Khan I., Katikaneni D.S., Han Q., Sanchez-Felipe L., Hanada K., Ambrose R.L., Mackenzie J.M., Konan K.V. (2014). Modulation of hepatitis C virus genome replication by glycosphingolipids and four-phosphate adaptor protein 2. J. Virol..

[26] Lomize A.L., Pogozheva I.D., Lomize M.A., Mosberg H.I. (2006). Positioning of proteins in membranes: a computational approach. Protein Sci..

[27] Lomize A.L., Pogozheva I.D., Mosberg H.I. (2011). Anisotropic solvent model of the lipid bilayer. 2. Energetics of insertion of small molecules, peptides, and proteins in membranes. J. Chem. Inf. Model..

[28] Lomize M.A., Pogozheva I.D., Joo H., Mosberg H.I., Lomize A.L. (2012). OPM database and PPM web server: resources for positioning of proteins in membranes. Nucleic Acids Res..

[29] Brooks B.R., Brooks C.L., Mackerell A.D., Nilsson L., Petrella R.J., Roux B., Won Y., Archontis G., Bartels C., Boresch S., Caflisch A., Caves L., Cui Q., Dinner A.R., Feig M. (2009). CHARMM: the biomolecular simulation program. J. Comput. Chem..

[30] Jo S., Kim T., Iyer V.G., Im W. (2008). CHARMM-GUI: a web-based graphical user interface for CHARMM. J. Comput. Chem..

[31] Lee J., Cheng X., Swails J.M., Yeom M.S., Eastman P.K., Lemkul J.A., Wei S., Buckner J., Jeong J.C., Qi Y., Jo S., Pande V.S., Case D.A., Brooks C.L., MacKerell A.D. (2016). CHARMM-GUI input generator for NAMD, GROMACS, AMBER, OpenMM, and CHARMM/OpenMM simulations using the CHARMM36 additive force field. J. Chem. Theory Comput..

[32] Wu E.L., Cheng X., Jo S., Rui H., Song K.C., Davila-Contreras E.M., Qi Y., Lee J., Monje-Galvan V., Venable R.M., Klauda J.B., Im W. (2014). CHARMM-GUI membrane builder toward realistic biological membrane simulations. J. Comput. Chem..

[33] Jorgensen W.L., Chandrasekhar J., Madura J.D., Impey R.W., Klein M.L. (1983). Comparison of simple potential functions for simulating liquid water. J. Chem. Phys..

[34] Kučerka N., Tristram-Nagle S., Nagle J.F. (2005). Structure of fully hydrated fluid phase lipid bilayers with monounsaturated chains. J. Membr. Biol..

[35] Kučerka N., Nagle J.F., Sachs J.N., Feller S.E., Pencer J., Jackson A., Katsaras J. (2008). Lipid bilayer structure determined by the simultaneous analysis of neutron and X-ray scattering data. Biophys. J..

[36] Phillips J.C., Hardy D.J., Maia J.D.C., Stone J.E., Ribeiro J.V., Bernardi R.C., Buch R., Fiorin G., Henin J., Jiang W., McGreevy R., Melo M.C.R., Radak B.K., Skeel R.D., Singharoy A. (2020). Scalable molecular dynamics on CPU and GPU architectures with NAMD. J. Chem. Phys..

[37] Huang J., Rauscher S., Nawrocki G., Ran T., Feig M., de Groot B.L., Grubmuller H., MacKerell A.D. (2017). CHARMM36m: an improved force field for folded and intrinsically disordered proteins. Nat. Methods.

[38] Nose S. (1984). A unified formulation of the constant temperature molecular-dynamics methods. J. Chem. Phys..

[39] Hoover W.G. (1985). Canonical dynamics: equilibrium phase-space distributions. Phys. Rev. A Gen. Phys..

[40] Humphrey W., Dalke A., Schulten K. (1996). VMD: visual molecular dynamics. J. Mol. Graph..

[41] Tubiana T., Carvaillo J.C., Boulard Y., Bressanelli S. (2018). TTClust: a versatile molecular simulation trajectory clustering program with graphical summaries. J. Chem. Inf. Model..

[42] Salentin S., Schreiber S., Haupt V.J., Adasme M.F., Schroeder M. (2015). PLIP: fully automated protein-ligand interaction profiler. Nucleic Acids Res..

[43] Rao C.S., Lin X., Pike H.M., Molotkovsky J.G., Brown R.E. (2004). Glycolipid transfer protein mediated transfer of glycosphingolipids between membranes: a model for action based on kinetic and thermodynamic analyses. Biochemistry.

[44] Rao C.S., Chung T., Pike H.M., Brown R.E. (2005). Glycolipid transfer protein interaction with bilayer vesicles: modulation by changing lipid composition. Biophys. J..

[45] West G., Nylund M., Peter Slotte J., Mattjus P. (2006). Membrane interaction and activity of the glycolipid transfer protein. Biochim. Biophys. Acta.

[46] Neumann S., Opacic M., Wechselberger R.W., Sprong H., Egmond M.R. (2008). Glycolipid transfer protein: clear structure and activity, but enigmatic function. Adv. Enzyme Regul..

[47] Zhai X., Malakhova M.L., Pike H.M., Benson L.M., Bergen H.R., Sugar I.P., Malinina L., Patel D.J., Brown R.E. (2009). Glycolipid acquisition by human glycolipid transfer protein dramatically alters intrinsic tryptophan fluorescence: insights into glycolipid binding affinity. J. Biol. Chem..

[48] Mattjus P. (2009). Glycolipid transfer proteins and membrane interaction. Biochim. Biophys. Acta.

[49] Kamlekar R.K., Gao Y., Kenoth R., Molotkovsky J.G., Prendergast F.G., Malinina L., Patel D.J., Wessels W.S., Venyaminov S.Y., Brown R.E. (2010). Human GLTP: three distinct functions for the three tryptophans in a novel peripheral amphitropic fold. Biophys. J..

[50] Ohvo-Rekila H., Mattjus P. (2011). Monitoring glycolipid transfer protein activity and membrane interaction with the surface plasmon resonance technique. Biochim. Biophys. Acta.

[51] Zou X., Chung T., Lin X., Malakhova M.L., Pike H.M., Brown R.E. (2008). Human glycolipid transfer protein (GLTP) genes: organization, transcriptional status and evolution. BMC Genomics.

[52] Mattjus P., Turcq B., Pike H.M., Molotkovsky J.G., Brown R.E. (2003). Glycolipid intermembrane transfer is accelerated by HET-C2, a filamentous fungus gene product involved in the cell-cell incompatibility response. Biochemistry.

[53] Kenoth R., Simanshu D.K., Kamlekar R.K., Pike H.M., Molotkovsky J.G., Benson L.M., Bergen H.R., Prendergast F.G., Malinina L., Venyaminov S.Y., Patel D.J., Brown R.E. (2010). Structural determination and tryptophan fluorescence of heterokaryon incompatibility C2 protein (HET-C2), a fungal glycolipid transfer protein (GLTP), provide novel insights into glycolipid specificity and membrane interaction by the GLTP fold. J. Biol. Chem..

[54] Kenoth R., Kamlekar R.K., Simanshu D.K., Gao Y., Malinina L., Prendergast F.G., Molotkovsky J.G., Patel D.J., Venyaminov S.Y., Brown R.E. (2011). Conformational folding and stability of the HET-C2 glycolipid transfer protein fold: does a molten globule-like state regulate activity?. Biochemistry.

[55] D'Angelo G., Rega L.R., De Matteis M.A. (2012). Connecting vesicular transport with lipid synthesis: FAPP2. Biochim. Biophys. Acta.

[56] Brodersen P., Petersen M., Pike H.M., Olszak B., Skov S., Odum N., Jorgensen L.B., Brown R.E., Mundy J. (2002). Knockout of Arabidopsis accelerated-cell-death11 encoding a sphingosine transfer protein causes activation of programmed cell death and defense. Genes Dev..

[57] Petersen N.H., McKinney L.V., Pike H., Hofius D., Zakaria A., Brodersen P., Petersen M., Brown R.E., Mundy J. (2008). Human GLTP and mutant forms of ACD11 suppress cell death in the Arabidopsis acd11 mutant. FEBS J..

[58] Moqadam M., Tubiana T., Moutoussamy E.E., Reuter N. (2021). Membrane models for molecular simulations of peripheral membrane proteins. Adv. Phys. X.

[59] Sommer B. (2013). Membrane packing problems: a short review on computational membrane modeling methods and tools. Comp. Struct. Biotech..

[60] Moradi S., Nowroozi A., Shahlaei M. (2019). Shedding light on the structural properties of lipid bilayers using molecular dynamics simulation: a review study. RSC Adv..

[61] Manni M.M., Tiberti M.L., Pagnotta S., Barelli H., Gautier R., Antonny B. (2018). Acyl chain asymmetry and polyunsaturation of brain phospholipids facilitate membrane vesiculation without leakage. Elife.

[62] Nylund M., Fortelius C., Palonen E.K., Molotkovsky J.G., Mattjus P. (2007). Membrane curvature effects on glycolipid transfer protein activity. Langmuir.

[63] Zhai X., Momsen W.E., Malakhov D.A., Boldyrev I.A., Momsen M.M., Molotkovsky J.G., Brockman H.L., Brown R.E. (2013). GLTP-fold interaction with planar phosphatidylcholine surfaces is synergistically stimulated by phosphatidic acid and phosphatidylethanolamine. J. Lipid Res..

[64] Nazeer M., Waheed H., Saeed M., Ali S.Y., Choudhary M.I., Ul-Haq Z., Ahmed A. (2019). Purification and characterization of a nonspecific lipid transfer protein 1 (nsLTP1) from Ajwain (Trachyspermum ammi) seeds. Sci. Rep..

[65] Madni Z.K., Tripathi S.K., Salunke D.M. (2020). Structural insights into the lipid transfer mechanism of a non-specific lipid transfer protein. Plant J..

[66] Shi Z., Wang Z.J., Xu H.L., Tian Y., Li X., Bao J.K., Sun S.R., Yue B.S. (2013). Modeling, docking and dynamics simulations of a non-specific lipid transfer protein from Peganum harmala L. Comput. Biol. Chem..

[67] Hunter N.H., Bakula B.C., Bruce C.D. (2018). Molecular dynamics simulations of apo and holo forms of fatty acid binding protein 5 and cellular retinoic acid binding protein II reveal highly mobile protein, retinoic acid ligand, and water molecules. J. Biomol. Struct. Dyn..

[68] Guo Y., Duan M., Yang M. (2019). The observation of ligand-binding-relevant open states of fatty acid binding protein by molecular dynamics simulations and a Markov state model. Int. J. Mol. Sci..

[69] Long D., Mu Y., Yang D. (2009). Molecular dynamics simulation of ligand dissociation from liver fatty acid binding protein. PLoS One.

[70] Ryan M.M., Temple B.R., Phillips S.E., Bankaitis V.A. (2007). Conformational dynamics of the major yeast phosphatidylinositol transfer protein sec14p: insight into the mechanisms of phospholipid exchange and diseases of sec14p-like protein deficiencies. Mol. Biol. Cell.

[71] Grabon A., Orlowski A., Tripathi A., Vuorio J., Javanainen M., Rog T., Lonnfors M., McDermott M.I., Siebert G., Somerharju P., Vattulainen I., Bankaitis V.A. (2017). Dynamics and energetics of the mammalian phosphatidylinositol transfer protein phospholipid exchange cycle. J. Biol. Chem..

[72] Nylund M., Mattjus P. (2005). Protein mediated glycolipid transfer is inhibited FROM sphingomyelin membranes but enhanced TO sphingomyelin containing raft like membranes. Biochim. Biophys. Acta.

[73] Wiener M.C., White S.H. (1992). Structure of a fluid dioleoylphosphatidylcholine bilayer determined by joint refinement of x-ray and neutron diffraction data. III. Complete structure. Biophys. J..

[74] Jiang T., Yu K., Hartzell H.C., Tajkhorshid E. (2017). Lipids and ions traverse the membrane by the same physical pathway in the nhTMEM16 scramblase. Elife.

[75] Pomorski T.G., Menon A.K. (2016). Lipid somersaults: uncovering the mechanisms of protein-mediated lipid flipping. Prog. Lipid Res..

[76] Perez C., Gerber S., Boilevin J., Bucher M., Darbre T., Aebi M., Reymond J.L., Locher K.P. (2015). Structure and mechanism of an active lipid-linked oligosaccharide flippase. Nature.

