# Supplementary material for: Ceramide-1-phosphate transfer protein promotes sphingolipid reorientation needed for binding during membrane interaction
Source: J Lipid Res. 2021 Nov 20;63(1):100151. doi: 10.1016/j.jlr.2021.100151 (PMC8953657; doi:10.1016/j.jlr.2021.100151)
Supplement: Supplemental Figures S1 and S2 [file mmc5.pdf]

Ceramide-1-Phosphate Transfer Protein Promotes Sphingolipid Reorientation Needed for Binding during Membrane Interaction

Yong-Guang Gao[1,*], Jeffrey McDonald[1], Lucy Malinina[1], Dinshaw J. Patel[2] and Rhoderick E. Brown[1,*]

[1]University of Minnesota, Hormel Institute, 801 16th Ave NE, Austin, MN 55912

[2]Structural Biology Program, Memorial Sloan Kettering Cancer Center, New York, NY

Supporting Information

Supporting Figures & Videos

Figure S1: Default settings for Optimized Charmm-Gui parameters. Default settings for all simulations were prepared on-line using the https://www.charmm-gui.org website.

Figure S2: Schematic of the proposed mechanism of CPTP interaction with a one side of a membrane containing C1P. Sphingolipid-free CPTP from the bulk aqueous milieu partitions into the lipid bilayer and then specifically binds C1P. The CPTP containing bound C1P at the bilayer interface releases into the bulk aqueous phase. Collisional interaction and docking of the CPTP containing bound C1P with a destination lipid bilayer enables release of the bound C1P into the bilayer.

Video S1: MD simulation focusing on α-3/α-4 helices connecting loop dynamics during POPC bilayer interaction. CPTP secondary structure is shown in ribbon representation. α-6 helix (lower right) is seen from a horizontal perspective (lower right); whereas the α-3/α-4 helices connecting loop is located within the red circle on the left. A significant downward dipping of the α-3/α-4 helices connecting loop towards the bilayer interface occurs at time 0-4 sec and 25-27 sec of the 50 sec video.

Video S2: MD simulation of wtCPTP interaction with C16-C1P embedded in a POPC bilayer. Note the relatively steady positioning of the C1P phosphate headgroup and the horizontal repositioning, i.e. 'wrapping' of the C1P hydrocarbon chains beneath the α-6 helix near the end of the simulation. CPTP secondary structure is shown in ribbon representation. Gold spheres represent phosphate groups in POPC. C16-C1P is shown in stick representation (hydrocarbon chains, cyan; nitrogen, blue, oxygen, red; and phosphate, orange). α-6 helix (lower right; end-on view), α-2 N-terminal region (lower center; horizontal view)penetrate shallowly into the bilayer and the α-3/α-4 helices connecting loop (lower left) shows close approach with the bilayer interface.

Video S3: MD simulation showing the lack wtCPTP engagement with C6-C1P embedded in a POPC bilayer. Note that the C6-C1P phosphate headgroup fails to remain engaged with α-6 helix (that contains the R155-R156 diArg motif) and shows no tendency for its hydrocarbons to horizontally reposition, i.e. 'wrap' closely beneath the α-6 helix and wanders deep into the POPC bilayer. CPTP secondary structure is shown in ribbon representation. C6-C1P is shown in stick representation (hydrocarbon chains, cyan; nitrogen, blue, oxygen, red; and phosphate, orange). POPC is not shown. α-6 helix (lower right; end-on view), α-2 N-terminal region (lower

center; horizontal view) penetrate shallowly into the bilayer; whereas the α-3/α-4 helices connecting loop (lower left) shows close approach with the bilayer interface region.

Video S4: MD simulation showing the lack of CPTP-R156L interaction with C16-C1P embedded in a POPC bilayer. Note the failure of the C1P phosphate headgroup to remain engaged with α-6 helix (that contains R156L) and the lack of horizontal repositioning, i.e. ‘wrapping’, of the C1P hydrocarbon chains beneath the mutated α-6 helix. CPTP secondary structure is shown in ribbon representation. C16-C1P is shown in stick representation (hydrocarbon chains, cyan; nitrogen, blue, oxygen, red; and phosphate, orange). POPC is not shown. α-6 helix (lower right), and α-2 N-terminal region (lower center) penetrate shallowly into the bilayer; whereas the α-3/α-4 helices connecting loop (lower left) shows close approach with the bilayer interface region.

Figure S1

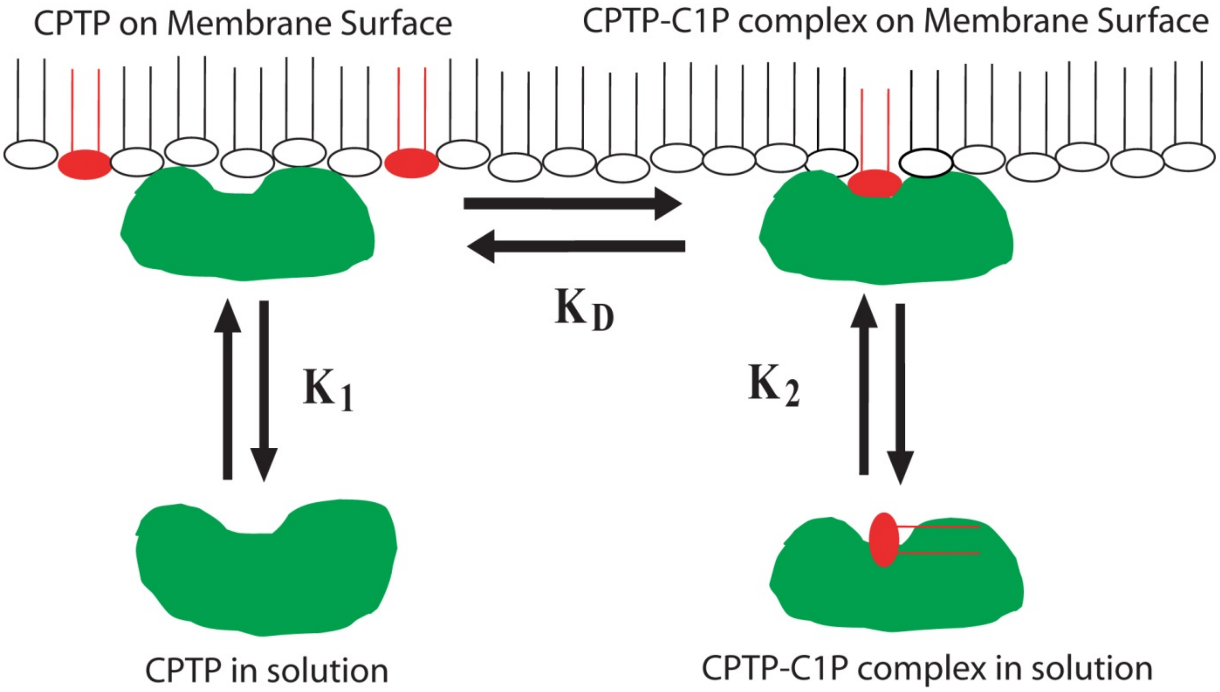

Figure S1. Schematic of the proposed mechanism of CPTP binding to vesicles containing C1P. Sphingolipid-free CPTP from the bulk aqueous milieu partitions into the lipid bilayer and then specifically binds C1P. The CPTP containing bound C1P at the bilayer interface is released into the bulk aqueous phase. Collisional interaction and docking of the CPTP containing bound C1P with the destination lipid bilayer enables release of the bound C1P into the bilayer.

Figure S2

Equilibration Input Notes:

```
!
! Setup Restraints for Protein and Lipids (see @liptype_restraint.str)
!
! Suggested Equilibration Scheme [Reducing Force Constants]
! (5 Cycles, 1 cycle = 50 - 100 ps )
!-----------------------------------------------------------------------
!        1 cycle    2 cycle    3 cycle    4 cycle    5 cycle    6 cycle
!-----------------------------------------------------------------------
! BB      10.0       5.0        2.5        1.0        0.5        0.1
! SC       5.0       2.5        1.0        0.5        0.1        0.0
! wforce   2.5       2.5        1.0        0.5        0.1        0.0
! tforce   2.5       2.5        1.0        0.5        0.1        0.0
! mforce   2.5       2.5        1.0        0.5        0.1        0.0
! ion     10.0       0.0        0.0        0.0        0.0        0.0
!-----------------------------------------------------------------------
```

Figure S2: Optimized Charmm-Gui parameters that served as default settings for all simulations prepared on-line using the https://www.charmm-gui.org website.
